# Clinicoradiological Profile of Lower Lung Field Tuberculosis Cases among Young Adult and Elderly People in a Teaching Hospital of Madhya Pradesh, India

**DOI:** 10.1155/2015/230720

**Published:** 2015-08-25

**Authors:** Saurabh Kumar Singh, Kamlesh Kumar Tiwari

**Affiliations:** Department of Pulmonary Medicine, Gajra Raja Medical College and Jayarogya Group of Hospitals, Gwalior, Madhya Pradesh 474009, India

## Abstract

*Aim*. To study the clinical and radiological features of lower lung field tuberculosis (LLFTB) in relation to the patients of nonlower lung field tuberculosis (non-LLFTB). *Material and Methods*. All the patients of lower lung field tuberculosis defined by the lesions below an arbitrary line across the hila in their chest X-rays were included in the study. Their sputum for acid fast bacilli, HIV, blood sugar, and other relevant investigations were performed. *Results*. The total of 2136 cases of pulmonary tuberculosis was studied. Among them 215 (10%) cases of patients were diagnosed as the case of lower lung field tuberculosis. Females (62%) were more commonly affected. Most common clinical feature in non-LLFTB was cough (69%) followed by fever (65%), chest pain (54.7%), and weight loss (54.4%). Chest X-ray showed predominance of right side (60.9%) in cases of LLFTB. The relative risk of having the LLFTB in diabetes patients, HIV seropositive patients, end stage renal disease patients, and patients on corticosteroid therapy was high. *Conclusion*. Lower lung field tuberculosis is not an uncommon entity. It is more common in diabetes, HIV positive, end stage renal disease, and corticosteroid treated patients. Clinical and radiological features are different from upper lobe tuberculosis patients.

## 1. Introduction

Tuberculosis is an ancient disease affecting mankind described as far back as 10,000 BC and it is still the major health problem worldwide. According to World Health Organization (WHO) 9 million people fell ill with TB in 2013, including 1.1 million cases among people living with HIV [1]. There were estimated 1.5 million deaths worldwide due to tuberculosis. WHO considered tuberculosis as one of the top killers of women of reproductive age group. The TB mortality rate has decreased 45% since 1990. In India 1.2 million new cases of TB were detected in the year 2013 with the estimated mortality of 0.2 million in non-HIV infected individuals.

Postprimary tuberculosis usually affects the upper lung field but the involvement of the lower lung field is also not uncommon. The lower lung field tuberculosis creates much confusion in the high tuberculosis burden region. It often masquerades as pneumonia, bronchiectasis, or bronchogenic carcinoma thus delaying the correct diagnosis. In the current AIDS/HIV epidemic there are increased incidences of middle and lower lung field tuberculosis.

Present study was conducted with the following aims (1) to study the incidence of LLFTB among patients with pulmonary tuberculosis; (2) to study the pattern of distribution of LLFTB and its relation with pulmonary tuberculosis; (3) to study factors influencing in the causation of LLFTB and its comparison with non-LLFTB patients; (4) to compare the clinicoradiological pattern between LLFTB and non-LLFTB.

## 2. Material and Methods

This study was conducted at Department of Pulmonary Medicine, Gajra Raja Medical College, Gwalior, India. It is a tertiary care teaching hospital. It was the prospective and descriptive study conducted during the time span of 24 months from January 2012 to January 2015. Ethical clearance was taken from the ethical committee of the college. All the patients of pulmonary tuberculosis were included in the study. Patients with pulmonary and extra pulmonary tuberculosis were considered as the case of pulmonary tuberculosis. Consent from the patients was taken before inclusion in the study.

History was taken in full detail regarding particulars of the patient and complaints including cough, weight loss, fever, hemoptysis, and anorexia, and history of contact with tuberculosis and history of any other systemic illness, diabetes mellitus, chronic liver diseases, asthma, chronic renal failure, and HIV were noted as well. Any relevant past history and personal history including dietary habits, smoking, alcohol, and other addictions were also taken. Diagnosis of tuberculosis was made by sputum for AFB examination by Ziehl-Neelsen technique. Those negative for sputum for AFB on two separate occasions were diagnosed as a case of sputum negative pulmonary tuberculosis on the basis of suggestive clinical and radiological findings which are nonresponsive to 2-3 weeks of antibiotics. These patients were considered as the confirm case of pulmonary tuberculosis if their clinical features improved on the administration of antitubercular drugs. Culture and sensitivity for acid fast bacilli were not performed. Patients who failed the treatment were referred to the centre with the culture facility.

**Figure 1:**
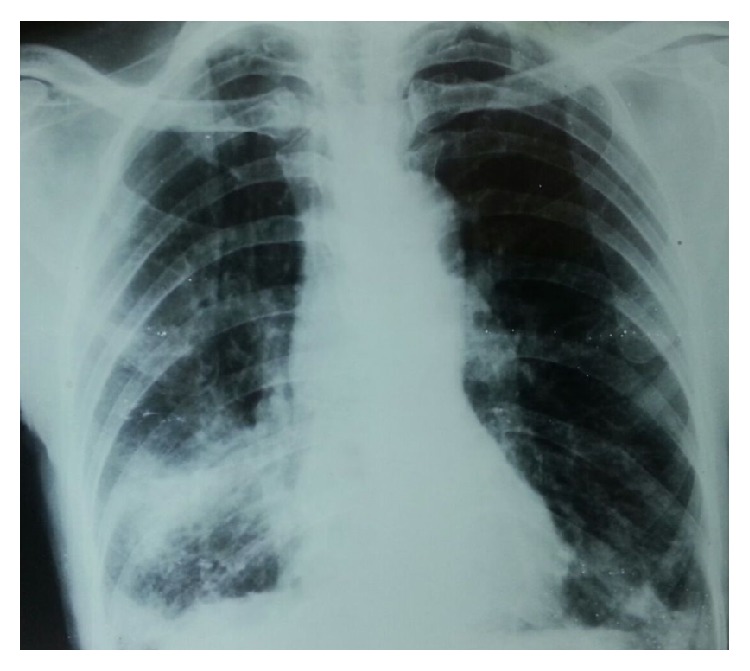
Chest X-ray with minimally advanced lesion.

**Figure 2:**
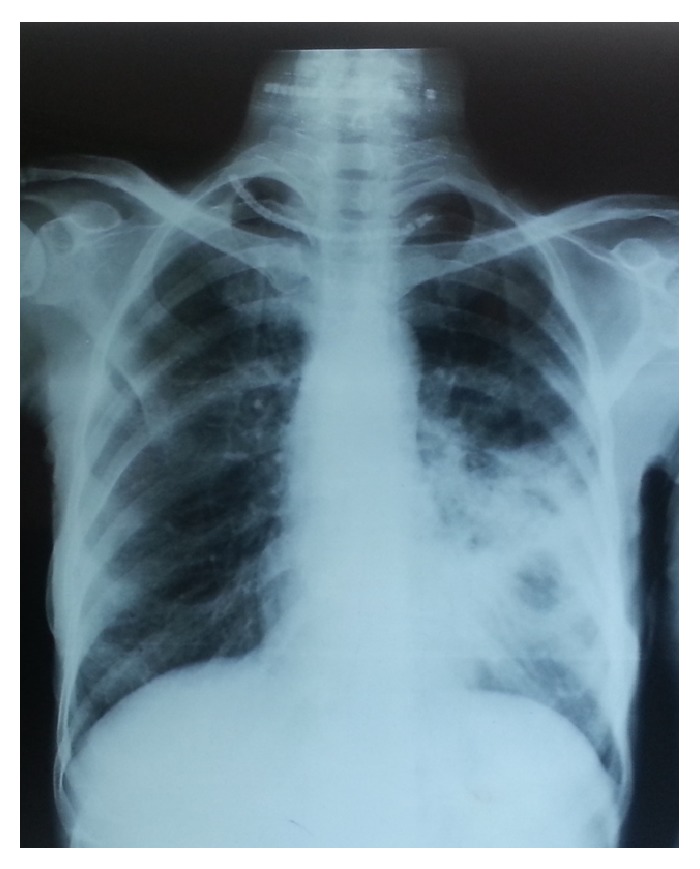
Chest X-ray showing moderately advanced lesion with cavity less than 4 cm in diameter.

**Figure 3:**
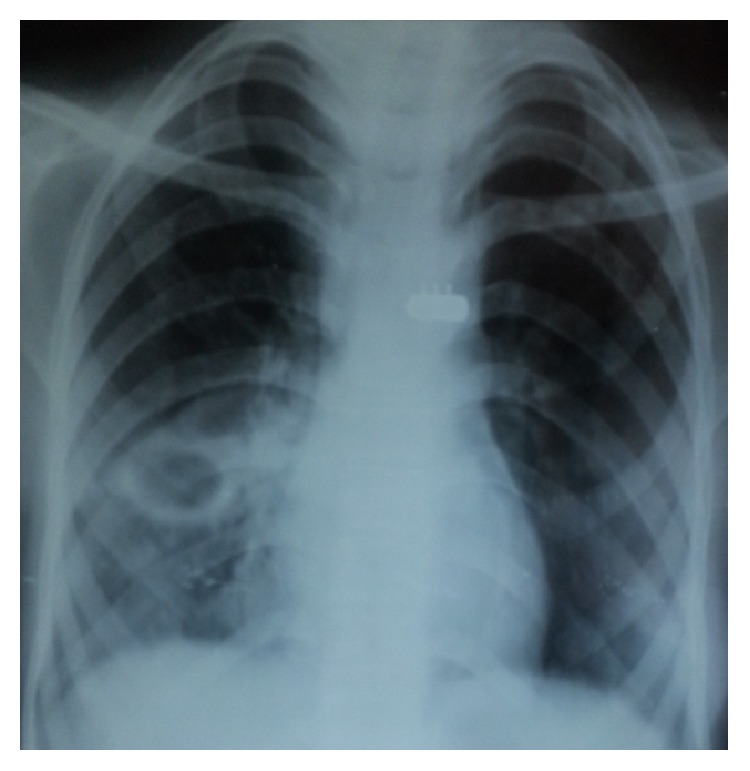
Chest X-ray showing far advanced lesion with cavity more than 4 cm in diameter.

Lower lung field tuberculosis on chest X-ray was defined as the area lying below the horizontal arbitrary line drawn across the hila on the chest X-ray (PA film). Parahilar region was considered in the lower lung fields [2]. Disease located in the lower lobes when lateral films were obtained. Record of the radiological reading in terms of consolidation, nodular opacity, and cavitation was also noted. Presence of pleural effusion, pneumothorax, fibrosis, and hilar lymphadenopathy was also noted. Recording of the abnormal shadows on chest X-ray includes the location using standard chest skiagram PA and lateral views; the extent of disease was classified as follows [3]:

1. Minimally advanced: lesion which was slight to moderate in density with no demonstrable cavitation, the total volume of lung on one side, present above the second chondrosternal junction, and spine of the fourth thoracic vertebra and no demonstrable cavity present (Figure 1).
2. Moderately advanced: disseminated lesions of slight to moderate density that may extend throughout the total volume of one lung or the equivalent in both lungs; dense or confluent lesions are limited in extent to one-third of the volume of one lung; and total diameter of cavitations, if present, must be less than 4 cm (Figure 2).
3. Far advanced: lesion more extensive than moderately advanced one (Figure 3).

Presence of pleural effusions, empyema, posttubercular bronchiectasis, and aspergilloma was regarded as complications of pulmonary tuberculosis.

Patients with the following characteristics were excluded from the study:

1. Patients less than 12 years of age were excluded.
2. Patients with upper lobe tuberculosis along with lower lobe involvement were not considered as the case of lower lobe tuberculosis.
3. Patients with pleural effusion without parenchymal involvement were excluded.

Diagnosis of diabetes was made according to WHO guidelines, that is, fasting plasma glucose ≥ 7.0 mmol/L (126 mg/dL) or 2 h plasma glucose ≥ 11.1 mmol/L (200 mg/dL). Strict control of sugar level was made during the treatment period. Patients were tested for HIV1 and HIV2 according to NACO guidelines [4]. Prolonged steroid intake was defined as steroid intake for more than three months.

All patients were treated with Directly Observed Treatment Short (DOTS) course for tuberculosis according to Revised National Tuberculosis Control Program (RNTCP) for India [5].

*Statistical Analysis*. Differences between the two groups were analyzed using the samples *t*-test (continuous variable) or the Chi square test (categorical variables). *p* value of <0.01 was taken as significant. Microsoft excel 2007 was used for the computation of statistics.

## 3. Results

The total of 2136 cases of pulmonary tuberculosis was studied. Among them 215 (10%) cases of patients were diagnosed as the case of lower lung field tuberculosis. Among lower lung field tuberculosis (LLFTB) 81 patients (38%) were males and 134 (62%) were females (Table 1). Mean age of male patients having lower lung field tuberculosis was 41.85 ± 14.14 years (12–70 years). The mean age of female patients having LLFTB was 35.64 ± 13.68 years (12–68 years). LLFTB affected females more frequently than males (*χ* ^2^ = 92.21, *p* = 0.001). Mean age of male and female non-LLFTB patients was 35.91 ± 13.17 and 37.98 ± 14.58, respectively. Age of the males in LLFTB group was significantly more than age of the males in non-LLFTB group. However, no such association was found in the females (Table 1).

Most common clinical feature in non-LLFTB was cough (69%) followed by fever (65%), chest pain (54.7%), and weight loss (54.4%), while in the patients with LLFTB cough (90.7%) was present in almost all the patients followed by fever (79.1%), hemoptysis (35.4%), and general malaise (38.1%). LLFTB patients presented significantly more with cough, fever, and hemoptysis. However, there was significantly low occurrence of chest pain and general malaise as compared to non-LLFTB group (Table 1). Sputum for* Mycobacterium tuberculosis* positivity in non-LLFTB and LLFTB was 1009 (52.52%) and 120 (55.8%). Though LLFTB have more number of sputum positive cases, that was not significant (*p* = 0.35).

Chest X-ray showed predominance of right side in cases of LLFTB and it was significant (*p* < 0.001). However, bilateral involvement was more common in non-LLFTB patients. Consolidation was the predominant finding in the chest X-ray in the LLFTB cases as compared to non-LLFTB (*p* < 0.001). Cavitation and hilar lymphadenopathy were more commonly found in the non-LLFTB cases as compared to LLFTB (*p* < 0.001).  Table 2 shows the finding of the chest X-ray in the two groups. In terms of grading the non-LLFTB showed mild, moderate, and severe grade as 642 (33.4%), 877, (45.7%) and 402 (20.9%), respectively, while the LLFTB showed mild, moderate, and severe grade as 45 (20.9%), 114 (53%), and 56 (26.1%), respectively (Table 3).

In the non-LLFTB group, 1172 (61%) patients were treated under category 1 DOTS while the rest were treated under category 2 DOTS; on the other hand in LLFTB group patients treated with category 1 DOTS were 140 (65.1%). Out of 215 patients in LLFTB, 190 (88.4%) patients were declared cured or treatment completed. Only 12 (5.5%) patients defaulted the treatment and 5 (2.3%) were declared as the failure cases. There was no significant difference with the non-LLFTB group. Eight LLFTB (3.7%) patients and 44 (2.3%) non-LLFTB patients had died during the study period. Three patients in LLFTB group had died due to tuberculosis itself and the cause was respiratory failure. Twenty-nine patients had died due to tuberculosis in non-LLFTB group and the cause was respiratory failure. Out of 29 patients, 5 had died due the sudden episode of massive hemoptysis and the rest died because of progression of pneumonia in which none other than* Mycobacterium* had been identified. Due to the non-TB related death in LLFTB two had died due to renal failure, two due to cancer, and one due to liver failure. Of the 15 non-TB related deaths in non-LLFTB group the cause of death was bacterial pneumonia in six, intestinal infection in five, myocardial infarction in two, and pancreatitis in two.

Diabetes, HIV, chronic renal failure with end stage renal disease, and corticosteroid therapy were found to be the significant risk factors in the development of LLFTB. There was significantly more incidence of LLFTB as compared to non-LLFTB cases. The relative risk of having the LLFTB was 4.95, 5.17, 3.25, and 2.13, respectively. Relative risk of having LLFTB was more than one with CRF, cancer, and cirrhosis but they were not significantly related to non-LLFTB cases (Table 4). Three patients in LLFTB group and 10 patients in non-LLFTB group were on more than 10 mg/day of steroid intake, while the rest of them were on more than 10 mg/day of steroid intake.

## 4. Discussion

Not only does lower lung field tuberculosis (LLFTB) differ from typical PTB radiologically but also it differs in clinical features as we found in our study. Reported prevalence of LLFTB without associated upper lobe disease ranges from 1 to 7% [2, 6–8]. In our study there were 10% of LLFTB cases out of the total tuberculosis patients. This was comparable to 10.6% prevalence reported from India [9]. In our study mean age of females was comparable in both groups. Mean age of males in LLFTB group was significantly more than that of non-LLFTB group. There is a wide gap in the reporting of the age group having lower lung field tuberculosis. Few studies reported mean age of patients of LLFTB to be 40 to 44 years [2, 8, 10]. However, other studies found that most of the cases of LLFTB occur in age group <20 years [6] while Zuber and Zaheer [9] reported that mean age of male patients having lower lung field tuberculosis was higher (35 years) than females (23 years). Female was more commonly affected with LFFTB in our study. Previous studies have also shown the same pattern. Study done in Taiwan had shown LLFTB, which was usually associated with endobronchial lesions, developing more frequently in women [11]. Other studies from India and Cameroon also showed higher incidence of LLFTB in females as compared to males [12, 13]. However, study done by Berger and Granada had showed higher incidence in males. The reasons for the sex-related difference were not known, though hypothesis was put that females have costal type of respiration which results in poor aeration of lower lobe and higher chances of tuberculosis [14]. The treatment outcome in both groups was comparable and it was consistent with other reported literatures [15].

Cough and hemoptysis were more common presenting feature in LLFTB patients as compared to the non-LLFTB patients in our study. The most common pathogenesis mechanism described for lower lung field is the ulceration of a bronchus by lymph node affected by tuberculosis with spillage of tuberculosis material into the bronchus. The ulceration might be the cause for cough and hemoptysis in our study. Cough was the main presenting feature in LLFTB patients in various other studies [6, 16]. Hemoptysis was noted in 35% of LLFTB cases as compared to 4.8% in non-LLFTB cases. Different studies had noted hemoptysis as an important clinical feature [2, 6]. Tripathy and Nanda had noted hemoptysis in nearly two-thirds of the cases [16]. Chest radiography showed right sided predominance in 61% of cases of LLFTB patients as compared to 37.2% in non-LLFTB cases. However, the bilateral involvement was more common in non-LLFTB cases. Literature search also showed the right sided predominance in LLFTB cases. Reported prevalence of right side lung involvement ranged from 54 to 73% [8, 10, 12]. Aktoğu et al. reported that the proportion of patients with isolated lower lung field TB was higher among females [17]. Bilateral lung involvement was seen in 14.9% of the cases of LLFTB in our study. Previous studies also had reported 10% of bilateral lesions in the LLFTB cases [6, 16]. Consolidation (56.7%) was the most frequent finding on chest X-ray in LLFTB cases in our study. Other findings seen on chest X-ray were infiltration (43.2%), cavitation (24.7%), hilar lymphadenopathy (4.6%), pleural effusion (92.3%), and pneumothorax (3.3%). These findings of chest X-ray differ significantly from non-LLFTB cases. In non-LLFTB cases infiltration followed by cavitation and consolidation was the common finding. However, the infiltration on chest X-ray was not significantly more than the patients of LLFTB. Study done by Berger and Granada stated that the findings in lower lung field tuberculosis differ significantly from those found in upper lobe disease and often resemble bacterial or viral pneumonia more than tuberculosis [7]. Pulmonary infiltration or consolidation tends to be more common in lower lung fields as compared to cavitation [18]. Other publications also supported consolidation as the common finding in the LLFTB cases [8, 13, 19]. Cavity was the commonest finding in the LLFTB in the study done by Vidyasagar et al. [12]. In our study 53% of the LLFTB cases had moderately advanced lesion as compared to 45.7% in non-LLFTB. It was not more significant in comparison to non-LLFTB cases. However, the mild lesion was present in 20.9% of LLFTB cases as compared to 33.4% in non-LLFTB cases. These results were more or less comparable to other studies [16, 20].

Relation with diabetes and tuberculosis is very old. Diabetes can predispose to tuberculosis by reactivation and diabetes may be caused by pulmonary tuberculosis due to insulin resistance [21]. The presenting feature of tuberculosis is usually not modified with diabetes but diabetes can modify the radiological features of tuberculosis in type and location on chest X-ray and the increased alveolar oxygen pressure in the lower lobes favors development of lower lobe disease in these groups [22]. In the present study the LLFTB was seen in 23.7% of cases of diabetes mellitus while the non-LLFTB was seen in 4.8% of diabetes mellitus cases. Other studies had also shown higher incidence of lower lung field tuberculosis in diabetics [2, 9]. Bacakoğlu et al. compared the patients with tuberculosis and diabetes seen during one-year period with an age- and sex-matched nondiabetic control group with tuberculosis. The presence of diabetes mellitus was found to not have an effect on patients' symptomatology, bacteriology results, tuberculin reaction, and localization of pulmonary infiltrates, except for lower lobe involvement in older patients [23]. In another study chest radiographs of 150 patients of pulmonary TB with diabetes and observed that 69 (46%) films showed the typical pattern involving upper zone, while 81 (54%) films showed the atypical pattern with lower lung field involvement [24]. There were other studies also that showed a higher incidence of lower lobe involvement among diabetic tubercular cases [11, 13, 25, 26], while the same trend of LLFTB in diabetes patients was not shown by others [27–29].

In patients of HIV the radiological features of tuberculosis differ from non-HIV case. Our study also confirmed these findings. There was significantly high number of LLFTB in HIV positive patients as compared to non-HIV positive cases. The relative risk of having the LLFTB in HIV positive cases was 5.17 (95% CI 3.12 to 8.58). In the patients of HIV it is common to have less number of upper lobe involvement as compared to lower lung involvement in tuberculosis cases [30]. LLFTB was reported to be more common in HIV positive as compared to non-HIV positive cases by Zuber and Zaheer [9]. Lack of consolidation and lack of cavities are the radiographic features that may be considered to define an atypical presentation since that is more common in HIV-seropositive than in HIV-seronegative patients [31]. HIV infected patients have high incidence of LLFTB compared to non-HIV infected persons.

In ESRD patients the clinical features of TB are usually atypical, mimicking the primary disease with lower lung or mid lung zone infiltrates [32]. In present study the relative risk of having LLFTB in ESRD was 3.25 (95% CI 1.4645 to 7.2081). LLFTB was present in 3.7% of ESRD cases as compared to 1.1% of non-LLFTB in ESRD. There was no significant association found in chronic renal failure cases (non-ESRD). In ESRD, pulmonary TB characterized by lymphadenopathy, pleural effusions, and zone infiltrates in the lower lung or mid lung on chest radiograph can be the presenting feature [33, 34].

Corticosteroids, through their immunosuppressive and anti-inflammatory effects, impair antibody formation and cell mediated immunity and thus predisposing patients to a variety of secondary infections including reactivation of latent tuberculous foci and reinfection with* Mycobacterium tuberculosis* [35]. In the present study patients on steroid therapy developed LLFTB (7.4%) and non-LLFTB (3.5%). There was significantly more number of cases of LLFTB in steroid treated cases and it is in compliance with other studies [19]. LLFTB was reported in 28.5% of steroid treated cases in Japan [36].

## 5. Conclusion

Lower lung field tuberculosis is a fairly common entity. It can be confused with the more common pneumonias located at that location causing the undue diagnosis and delay in the treatment. It affects females more commonly as compared to males and tuberculosis should be looked in females with lower lung field lesions. It differs from upper lung field tuberculosis in clinical and radiological features. Cough and hemoptysis are the most frequent presentation of LLFTB as compared to non-LLFTB cases. On chest X-ray right sided involvement is the most commonly encountered presentation. Consolidation and infiltration are the most frequent finding on chest X-ray in LLFTB cases in comparison to cavitation seen in non-LLFTB cases. Patients with diabetes, HIV, and end stage renal disease cases and persons on corticosteroid frequently present with LLFTB. Short course antitubercular chemotherapy is quite an effective mode of treatment in treating LLFTB.

## 6. Limitations

Bronchoscopy was not performed in all the cases which may be considered important in lower field tuberculosis patients. In HIV positive patients CD4 level was not taken into consideration and it is well known that pattern of chest X-ray in pulmonary tuberculosis patients is different in high and low level of CD4 level. In our study glycated hemoglobin (HbA1c) was also not measured which is the measure of the diabetes control. Therefore, we were not able to differentiate whether the control diabetics have more chances of having LLFTB than noncontrol diabetics. The study was conducted in tertiary care hospital where patients are tending to be more terminally ill. Therefore, further studies in the general population will provide more substantial data on the LLFTB.

## Figures and Tables

**Table 1:** Clinical characteristic in lower lung field tuberculosis (LLFTB) and non-LLFTB cases.

	LLFTB (215)	Non-LLFTB (1921)	*p* value
Sex, male/female	81/134	1348/573	<0.001
Age (years)			
Male	41.85 ± 14.14	35.91 ± 13.17	0.001
Female	35.64 ± 13.68	37.98 ± 14.58	0.14
Cough	195 (90.7%)	1336 (69.5%)	<0.001
Dry	26 (12.1%)	307 (16%)	
Productive	169 (78.6%)	1029 (53.5)	
Fever	170 (79.1)	1248 (65%)	<0.001
Hemoptysis	76 (35.4%)	68 (4.8%)	<0.001
Chest pain	72 (33.5%)	1050 (54.7%)	<0.001
Weight loss	101 (47%)	1045 (54.4%)	0.04
General malaise	82 (38.1)	936 (48.7%)	0.004
Night sweat	30 (14%)	390 (20.3%)	0.026
Anemia	114 (53%)	1092 (56.9%)	0.28

**Table 2:** Comparison of radiological features in two groups.

	Lower lung field tuberculosis (215)	Nonlower lung field tuberculosis (1921)	*p* values
Right side	131 (60.9%)	714 (37.2%)	<0.001
Left side	52 (24.2%)	633 (33%)	0.009
Bilateral involvement	32 (14.9%)	574 (29.9%)	<0.001
Infiltration	93 (43.2%)	889 (46.3%)	0.399
Consolidation	122 (56.7%)	749 (39.0%)	<0.001
Cavitation	53 (24.7%)	920 (47.9%)	<0.001
Hilar lymphadenopathy	10 (4.6%)	442 (23.0%)	<0.001
Pleural effusion	5 (2.3)	384 (20.0%)	<0.001
Pneumothorax	7 (3.3%)	52 (2.7%)	0.64
Miliary tuberculosis	0 (0%)	52 (2.7%)	0.008

**Table 3:** Radiological grading in lower lung field tuberculosis patients (LLFTB) and nonlower lung field tuberculosis (non-LLFTB) patients.

Extent	LLFTB (*n* = 215)	Non-LLFTB (*n* = 1921)	*p* value
Mild advanced	45 (20.9%)	642 (33.4%)	<0.001
Moderately advanced	114 (53%)	877 (45.7%)	0.04
Far advanced	56 (26.1%)	402 (20.9%)	0.08

**Table 4:** Association of different diseases with lower lung field tuberculosis (LLFTB) and non lower lung field tuberculosis (non- LLFTB) patients.

Disease	LLFTB (%)	non- LLFTB (%)	*p* value	RR (95% CI)
Diabetes	51 (23.7)	92 (4.8%)	<0.001	4.95 (3.6263 to 6.7652)
HIV	22 (10.2%)	38 (2%)	<0.001	5.17 (3.1193 to 8.5783)
CRF (Non ESRD)	22 (10.2)	165 (8.6)	0.41	1.19 (0.7812 to 1.8167)
End stage Renal Disease	8 (3.7)	22 (1.1)	0.004	3.25 (1.4645 to 7.2081)
Cancer	14 (6.5)	82 (4.3)	0.13	1.52 (0.8783 to 2.6331)
Prolonged oral Steroid Therapy	16 (7.4)	67 (3.5)	0.005	2.13 (1.2599 to 3.6136)
Pregnancy	17 (12.7)	78 (13.6)	0.78	0.93 (0.5711 to 1.5209)
Cirrhosis	12 (5.6)	99 (5.2)	0.79	1.08 (0.6050 to 1.9388)
